# Supplementary material for: Complexome profiling of the Chlamydomonas psb28 mutant reveals TEF5 as an early PSII assembly factor
Source: Plant Cell. 2025 Mar 17;37(6):koaf055. doi: 10.1093/plcell/koaf055 (PMC12164590; doi:10.1093/plcell/koaf055)
Supplement: koaf055_Supplementary_Data [file koaf055_supplementary_data.zip › psb28_interactive.zip!/plots/intensity/qualityPlots/peptides/_Cre13.g573400.t1.2.html]

### Cre13.g573400.t1.2

Trivial name:   
SeqCoverage [median]: 16.10  
Mass: 24.71 kDa  
  
 

| Sequence | MissedCleavage | Mass [Da] | wtIntensity | muIntensity | Score |
| --- | --- | --- | --- | --- | --- |
| AAIRVPFTR | 1 | 1029.61 | 29269.96 | 20535.28 | 130.27 |
| IIPTALK | 0 | 754.50 | 352732.35 | 188617.83 | 100.07 |
| VVDTTNDASSGAGWTIGENGLPK | 0 | 2288.09 | 4776.80 | 5421.57 | 98.96 |
|  |  |  | 386779.1 | 214574.7 |  |
